# AIF Downregulation and Its Interaction with STK3 in Renal Cell Carcinoma

**DOI:** 10.1371/journal.pone.0100824

**Published:** 2014-07-03

**Authors:** Shengqiang Xu, Hongjin Wu, Huan Nie, Lei Yue, Huadong Jiang, Sheng Xiao, Yu Li

**Affiliations:** 1 School of Life Science and Technology, Harbin Institute of Technology, Harbin, China; 2 Department of Pathology, Brigham and Women’s Hospital, Harvard Medical School, Boston, Massachusetts, United States of America; Center for Molecular Biotechnology, Italy

## Abstract

Apoptosis-inducing factor (AIF) plays a crucial role in caspase-independent programmed cell death by triggering chromatin condensation and DNA fragmentation. Therefore, it might be involved in cell homeostasis and tumor development. In this study, we report significant AIF downregulation in the majority of renal cell carcinomas (RCC). In a group of RCC specimens, 84% (43 out of 51) had AIF downregulation by immunohistochemistry stain. Additional 10 kidney tumors, including an oxyphilic adenoma, also had significant AIF downregulation by Northern blot analysis. The mechanisms of the AIF downregulation included both *AIF* deletion and its promoter methylation. Forced expression of AIF in RCC cell lines induced massive apoptosis. Further analysis revealed that AIF interacted with STK3, a known regulator of apoptosis, and enhanced its phosphorylation at Thr180. These results suggest that AIF downregulation is a common event in kidney tumor development. AIF loss may lead to decreased STK3 activity, defective apoptosis and malignant transformation.

## Introduction

Renal cell carcinoma (RCC) accounts for approximately 90% of all kidney malignancies with rising incidence over the last decades [1], [2]. Nephrectomy is effective in removing early tumors. However, when metastasis occurs, RCC is very difficult to treat and has poor prognosis. In spite of recent developments in targeted therapy, which significantly extend median survival, most patients still succumb to cancer.

Apoptosis-inducing factor (AIF) was initially identified as a death effector involved in caspase-independent apoptosis pathway [3]–[5]. Full length AIF is synthesized in cytoplasm as a precursor (67 kDa). When transported into mitochondria, the precursor is processed by cutting the first 34 amino acids and becomes a 62 kDa mature AIF (AIFmit) that acts as an NADH oxidase [6], [7]. In normal cell, AIF anchors to the inner mitochondrial membrane and plays a vital function in maintaining mitochondrial structure and respiratory chain. In response to apoptotic stimuli, AIF is cleaved by calpains or cathepsins to yield a 57 kDa truncated form that translocates to the nucleus to induce DNA condensation and large-scale fragmentation in the presence of endonuclease G (EndoG) [8]–[11]. Recent studies on necroptosis, a regulated type of necrosis, have revealed that AIF plays several roles in alkylating DNA damage agent N-methyl-N′-nitro-N′-nitrosoguanidine-mediated necroptosis [12]. AIF is reported to interact with histone H2AX and form a DNA-degrading complex with cyclophilin A [13], [14]. In addition to its role in cell death, AIF is also involved in cell survival. For example, AIF-deficient cells have incomplete complex I and are susceptible to peroxide-induced apoptosis [15]. AIF also participates in maintaining the transformed state of colon cancer cells [16].

Serine/threonine-protein kinase 3 (STK3, also called MST2) is a pro-apoptotic cytoplasmic kinase belonging to the Ste-20 kinase family. STK3 and its family member STK4 (MST1) are the core elements of mammalian Hippo pathway that controls cell development, proliferation, apoptosis, and various stress responses. In the Hippo signaling pathway, STK3 can be activated by binding to the adaptor Salvador Homolog 1(WW45). Activated STK3 can then further activate its downstream kinase, Large Tumor Suppressor Kinase (LATS), which phosphorylates both Yes-Associated Protein (YAP) and Tafazzin (TAZ). Phosphorylation allows YAP and TAZ to be retained by 14-3-3 protein in the cytoplasm, so that they cannot translocate to nucleus to regulate gene transcription [17], [18]. STK3 is also involved in several pro-apoptotic processes. Ras association domain-containing protein 1 (RASSF1A) induces apoptosis by both releasing STK3 from Raf1 binding and enhancing STK3 interaction with LATS1 [19]. In the MST-FOXO signaling pathway that mediates oxidative stress-induced neuronal death, STK3/STK4 (MST2/MST1) phosphorylate forkhead box protein O3 (FOXO3) [20]. The phosphorylation of FOXO3 disrupts its interaction with 14-3-3 proteins and promotes FOXO3 translocation to the nucleus. Overexpression of STK3 or STK4 (MST1) induces a caspase-independent morphological change characteristic of apoptosis. These cells are also more sensitive to death receptor-mediated apoptosis than their wild-type counterparts [21]. STK4 activation can also induce H2B phosphorylation and chromatin condensation via a caspase-dependent pathway [22], [23]. In addition, multiple apoptotic agents including staurosporine, Fas ligand, and heat shock induce STK3 activation in cultured mammalian cells [24]. Although high levels of STK3 have been observed in human kidney, its roles in kidney has not been described thoroughly [25].

In this study, we show that AIF is significantly down regulated in RCC and AIF interacts with STK3. Furthermore, overexpression of AIF in RCC results in STK3 activation and cell death. It should be noted that AIF is located on chromosome X. Interestingly, kidney tumors are often more common in male (female to male ratio is 1∶1.6 for RCC) and male patients typically have higher possibility of metastatic spread and worse prognosis, particularly in clear cell renal cell carcinoma (CCRCC) [26], [27]. These findings indicate that genes on chromosome X play a role in RCC development, and the function of AIF in tumorigenesis is related to its regulation on STK3.

## Materials and Methods

### Tumor specimens

Renal cell carcinoma tissue specimens including 51 formalin-fixed and paraffin embedded samples, and 37 liquid nitrogen-frozen samples with the paired normal adjacent tissues were obtained from the First Affiliated Hospital, Harbin Medical University from 2005 to 2009. Clinicopathologic data are summarized in Table S1. Tumor types and stages were assigned according to UICC criteria. All the clinical samples were obtained from patients with written informed consent. The usage of patients’ samples was approved by the Medical Ethics and Human Clinical Trial Committee of the First Affiliated Hospital, Harbin Medical University.

### Cell culture

Human Embryonic Kidney 293 cells (HEK293) were grown in Dulbecco’s modified Eagle’s medium (Corning Cellgro, USA) supplemented with 10% fetal bovine serum (FBS) (GIBCO, USA) and 200 mM L-glutamine (Sigma, USA). RCC cell lines Caki-1 (HTB-46) and 786-O (CRL-1932) were grown in RPMI 1640 medium (Corning Cellgro, USA) with 10% FBS and 200 mM L-glutamine. All cells were cultured at 37°C in humidified incubator with 5% CO_2_.

### Plasmid construction and transfection

Total RNA was extracted from HEK293 cell using TRIzol reagent (Invitrogen, USA). First strand cDNA was synthesized using cDNA Synthesis Kit (Thermo, USA). *AIF* transcript variant 1 and *STK3* were amplified and cloned into *pcDNA3.1* (Invitrogen, USA), *pGEX-6p-1* (GE Healthcare, USA) or *pCMV-FLAG* (*pCMV-HA* was from Clontech, with HA tag replaced by FLAG). Transient transfections were performed using Lipofectamine-2000 reagent (Invitrogene, USA) according to manufacturer’s protocol.

### AIF Northern blot analysis

A tumor tissue array (BD Clontech Cancer Profiling Array II) was hybridized with a p32-labeled *AIF* cDNA probe, detected by exposing to an X-ray film. p32-labeled *ATN* cDNA probe was also hybridized as control. Hybridization and washing conditions were performed according to manufacturer’s protocol.

### Immunohistochemistry and Western blot analysis

Tumor sections were heat-treated to retrieve antigen, blocked in 3% BSA in PBST for 30 min and incubated with AIF antibody (Santa Cruz Biotech, sc-55519, USA; 1∶200 dilution in PBST) overnight at 4°C [28]. HRP-goat anti-mouse secondary antibody (1∶1000 dilution in PBS) (Santa Cruz, USA) was added for 1 h and detected by diaminobenzidine (Sigma, USA). Cells were counterstained with hematoxylin. Antibody testing is showed on Fig. S1.

For western blot analysis, cryostat sliced frozen tissue were lysed in RIPA buffer with PMSF and protease inhibitor cocktails (Thermo, USA). Crude protein lysates were used for analysis. All the western blot tests were repeated at least three times. Quantity One software was used in the quantification of western blot data.

### GST pull-down assay and immunoprecipitation

For GST pull-down assay and immunoprecipitation, cultured cells were harvested using non-denaturing lysis buffer with PMSF and protease inhibitor cocktails [29], [30]. The following antibodies were used: anti-AIF antibody (Abcam; ab1998), anti-STK3 and phospho-STK3 antibodies (Cell Signaling; #3952 and #3681), anti-V5 tag and anti-FLAG tag antibodies (Abcam; ab27671 and ab45766), anti-Caspase-3 and anti-actin antibodies (Santa Cruz; sc-56046 and sc-1616).

GST and GST-AIF plasmids were transformed into E. coli BL21 (DE3) and cultured at 37°C until OD_600_ reached 1.0. At this point, IPTG was added (final concentration of 0.25 mM) and cells were then clutured at 16°C for 6 h. The GST or GST-AIF fusion proteins were extracted with lysis buffer and sonicated on ice. After centrifuge, the supernatant of E.coli lysates was immobilized onto glutathione-sepharose 4B resin (GE, USA) at 4°C for 2 h. For the GST pull-down assay, the immobilized GST or GST-fusion proteins were incubated with cell lysates at 4°C for 2 h. After being washed for six times, proteins remaining on the sepharose beads were resuspended with SDS sample buffer, resolved with SDS-PAGE and stained by Coomassie Brilliant Blue G-250. All interested bands were analyzed by Mass-spectrometry.

For immunoprecipitation, *pcDNA3.1-AIF-V5* or *pCMV-Flag-STK3* were transfected into Caki-1 cells separately. Cells were harvested with lysis buffer 12 h later. After pre-clean, cell lysates were incubated overnight with V5 or FLAG antibody. Immune complex were precipitated by adding protein A beads (GE, USA) and detected by western blot.

### Fluorescence in situ hybridization (FISH)

Dual-color FISH was performed as described [31]. BAC DNA clone RP3-438D16 (BACPAC Resources Center, USA) containing the entire *AIF* genome sequence was labeled with rhodamine using BioPrime DNA Labeling System (Life Technologies, USA). Chromosome X centromeric probe (Metasystems, USA) was used as a control probe. Hybridization signals were counted in 100 morphologically intact nuclei for each case using a Leica DMRB fluorescence microscope equipped with a 100× oil immersion objective. *AIF* allelic reduction (or deletion) was scored if *AIF* signals were less than the control centromere probe in a cell. The cutoff value for an *AIF* deletion established from non-neoplastic kidney tissue for this probe was 5%.

### AIF promoter bisulfite-PCR and sequencing

Three pairs of overlapping bisulfite-specific PCR (BSP) primer sets, covering the fragment from −635 to +860, were designed according to the instructions of Bisulfite Primer Seeker Program (www.zymoresearch.com/tools/bisulfite-primer-seeker). Primer sequences are showed in Table S2. DNA samples were treated with EZ DNA Methylation-Gold Kit (Zymo Research Corp., USA) for bisulfite conversion. PCR were performed with the corresponding primer sets 30 cycles each. PCR products were cloned into pMD18-T Vector (Takara, Japan). Ten clones from each case were selected randomly for sequencing using BigDye Direct Cycle Sequencing Kit (Applied Biosystems, USA). Applied Biosystems 3500 and 3500 Series Data Collection Software were used for DNA sequencing and data analysis.

### Apoptosis analysis

Cell apoptosis was assessed by Annexin V-FITC Apoptosis Detection Kit I (BD Biosciences). Briefly, cells (1×10^6^/well) were plated in six-well plates and cultured overnight. After transfection with *pcDNA3.1-AIF-V5*, cell were collected at different time points (from 8 h to 16 h), and stained with Annexin V-FITC/PI followed by FACS analysis.

### Statistics

The statistical significance of AIF expression difference between RCC and normal kidney tissues was determined by Student’s T test. A value of p<0.05 was considered statistically significant.

## Results

### AIF is down regulated in RCC

**Figure 1:**
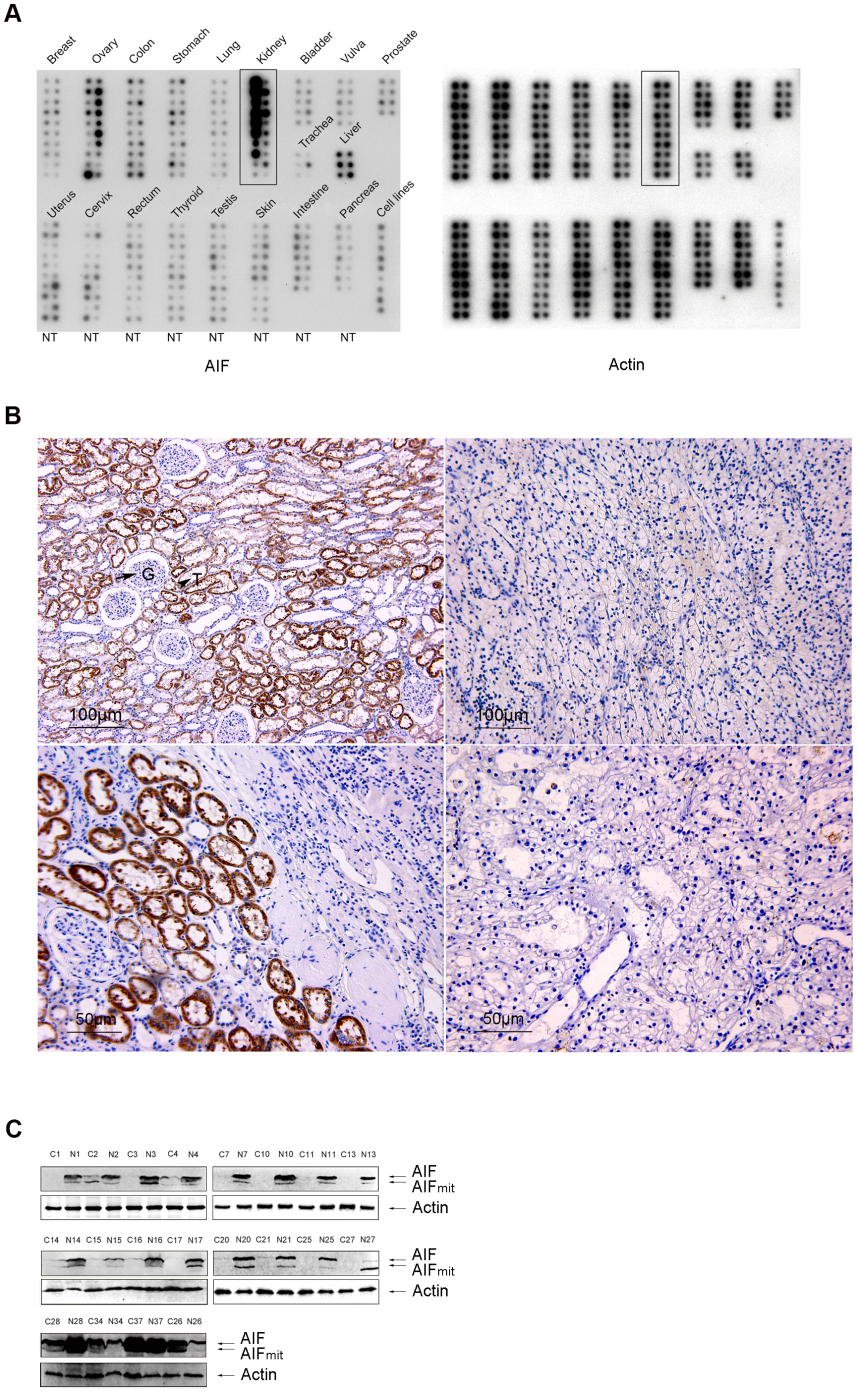
Downregulation of AIF in kidney tumors. (A) A northern blot analysis of 19 types of tumors was performed with an AIF-specific cDNA probe. Significant AIF downregulation was detected in various kidney tumors (from top to bottom: RCC, oxyphillic adenoma, RCC, RCC, transitional cell carcinoma, CCRCC, transitional cell carcinoma, RCC, epithelial nephroblastoma, and cancer of left kidney). Equal RNA loading was verified by hybridization with actin. N, normal tissue; T, Tumor tissue. (B) Immunohistochemistry showed AIF downregulation in RCCs. AIF was expressed in normal renal tubule (arrowhead) but not in glomeruli (arrow) (up left panel), and no AIF signals were seen in an RCC (up right panel). Assay of AIF expression in a normal kidney with partial adjacent cancer tissue and paired RCC tissue from the same patient are shown on bottom panel. Strong staining of AIF was observed in normal kidney part (lower left panel) compared to very weak staining in cancer part (lower right panel). (C) Western blot analyses of 20 CCRCCs with paired normal tissues showed no expression of AIF or significant AIF downregulation in 17 CCRCCs. Only three cases (#34, #37, #26) didn’t show AIF downregulation. N, normal tissue; C, cancer tissue.

A northern blot analysis of 154 tumors and corresponding normal tissues (BD Clontech Cancer Profiling Array II) showed that, among 19 tissue types, *AIF* is expressed highest in normal kidney. *AIF* downregulation was detected in various kidney tumors including a benign renal oxyphilic adenoma, RCCs, and transitional cell carcinomas (Fig. 1A). No significant AIF downregulation was detected in the remaining 18 tumor types. We studied AIF protein expression by immunohistochemistry on 51 cases of kidney tumors including 45 clear cell RCC (CCRCC), 4 papillary RCCs, a chromophobe RCC and a renal oncocytoma. Among 51 tumors, 37 cases had matching normal kidney tissue specimens. While all normal kidney had strong AIF signals in the convoluted tubules (++ or +++), none or decreased AIF (− or −/+) was detected in 43 RCCs. Moreover, 38 out of 45 CCRCC samples showed decreased AIF expression compared with the normal ones (Fig. 1B; Table 1 and Table S3). In addition, no significant associations between AIF expression and tumor characteristics (size, metastasis, age and gender) were detected in CCRCCs (Fisher’s exact test, p>0.05) (Table S4). Since CCRCC is the most common type representing around 80% of all RCC in most series [32], [33], we focused on AIF expression in CCRCCs. To confirm the immunohistochemistry findings, we randomly chose 20 of these 45 CCRCC tumors for a western blot analysis, significant AIF downregulation was detected in 17 out of 20 cases (Fig. 1C). Overall, these findings show that AIF downregulation is a common event in RCC, especially in CCRCC.

**Table 1:** AIF expression in clinical samples of kidney tumors.

		AIF expression level	
Feature	n	+∼+++	−∼−/+*
**Normal**	37	37 (100%)	0 (0%)
**RCC**	51	8 (15.7%)	43 (84.3%)
CCRCC	45	7 (15.6%)	38 (84.4%)
Others **	6	1 (16.7%)	5 (83.3%)

*+++ : >50% cells stained and brown in tissue; ++: 20–50% cells stained and snuff color in tissue; +: mild to moderate staining of 5–20% cells in tissue; − /+ : <5% of cells stained and faint in tissue; −: negative, non-stained cells in tissue;

*P* value between Normal and Tumor group is less than 0.001.

**Others contain 4 papillary RCC, 1 renal oncocytoma and 1 chromophobe RCC.

### AIF genome DNA deletion and promoter methylation in RCC

**Figure 2:**
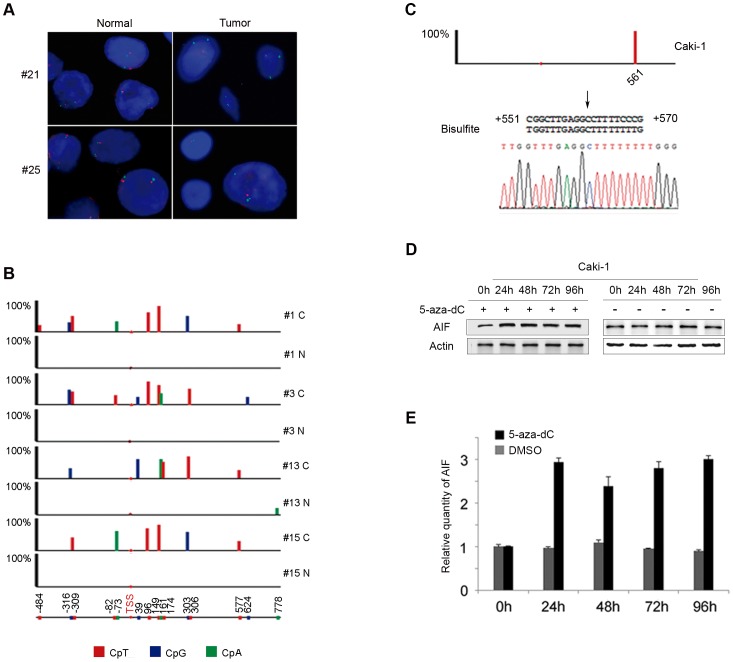
*AIF* deletion and promoter methylation in kidney tumors. (A) FISH analyses of 2 RCCs showed loss of AIF (red) as compared to a control chromosome X centromere probe (green). (B) Bisulfite sequencing analyses of *AIF* promoter region in 4 paired male CCRCCs had aberrant methylation patterns including CpA (green), CpT (red) and CpG (blue). Location of each methylation point is noted at the bottom. Height of columns reflects the frequency detected in CCRCCs. (C) Bisulfite sequencing analyses of *AIF* promoter region in RCC cell line Caki-1. (D & E) Caki-1 cells were treated with demethylation agent 5-aza-dC at 5 µM or vehicle DMSO for various periods of time; and western blot was performed with antibodies to AIF and actin. AIF levels were quantitated by normalization to actin.

To detect potential AIF mutations, AIF was amplified by exon-by-exon PCR in 37 pairs of RCC/normal tissues and PCR products were analyzed by SSCP (PCR primer sets are showed in Table S5). No significant AIF mutations were detected in these tumors. We performed AIF FISH analysis using a BAC clone containing the entire *AIF* genome sequence, and identified AIF allelic reduction in 2 out of 11 tumors (Fig. 2A).

Since *AIF* genome deletion was not accountable for most RCCs with AIF downregulation, we studied the methylation status of *AIF* promoter region. A 1495 bp region (−635 to +860) covering 70 CpG dinucleotides was selected for methylation studies. To circumvent the interference of hypermethylated loci on the inactive X-chromosome in females, 4 pairs of male CCRCC samples with downregulated AIF (no apparent *AIF* loss) and a male RCC cell line Caki-1 were selected for *AIF* promoter methylation studies. Bisulfite sequencing showed a complicated DNA methylation profiling (Fig. 2B). Multiple DNA methylation sites, including non-CpG methylation, were detected in all 4 tumor samples. Most common methylation sites in RCC were, in descending order: 96 (CpT), 149 (CpT), −316 (CpG), −309 (CpT), +303 (CpG), and +306 (CpT) (Fig. S2). In addition, we detected a unique constant CpT methylation at +561 in Caki-1 cell as shown in Figure 2C.

To study whether *AIF* promoter methylation affects *AIF* expression, Caki-1 cells were treated with a DNA demethylating agent 5-aza-2′-deoxycytidine (5-aza-dC) at 5 µM for 24 h, which led to almost three-fold increase of AIF as compared to the vehicle-treated controls (Fig. 2D and 2E). Taken together, these results demonstrate that aberrant *AIF* promoter methylation is a common event in RCC that may play a major role in AIF downregulation in these tumors.

### AIF overexpression induces apoptosis in RCC cells

**Figure 3:**
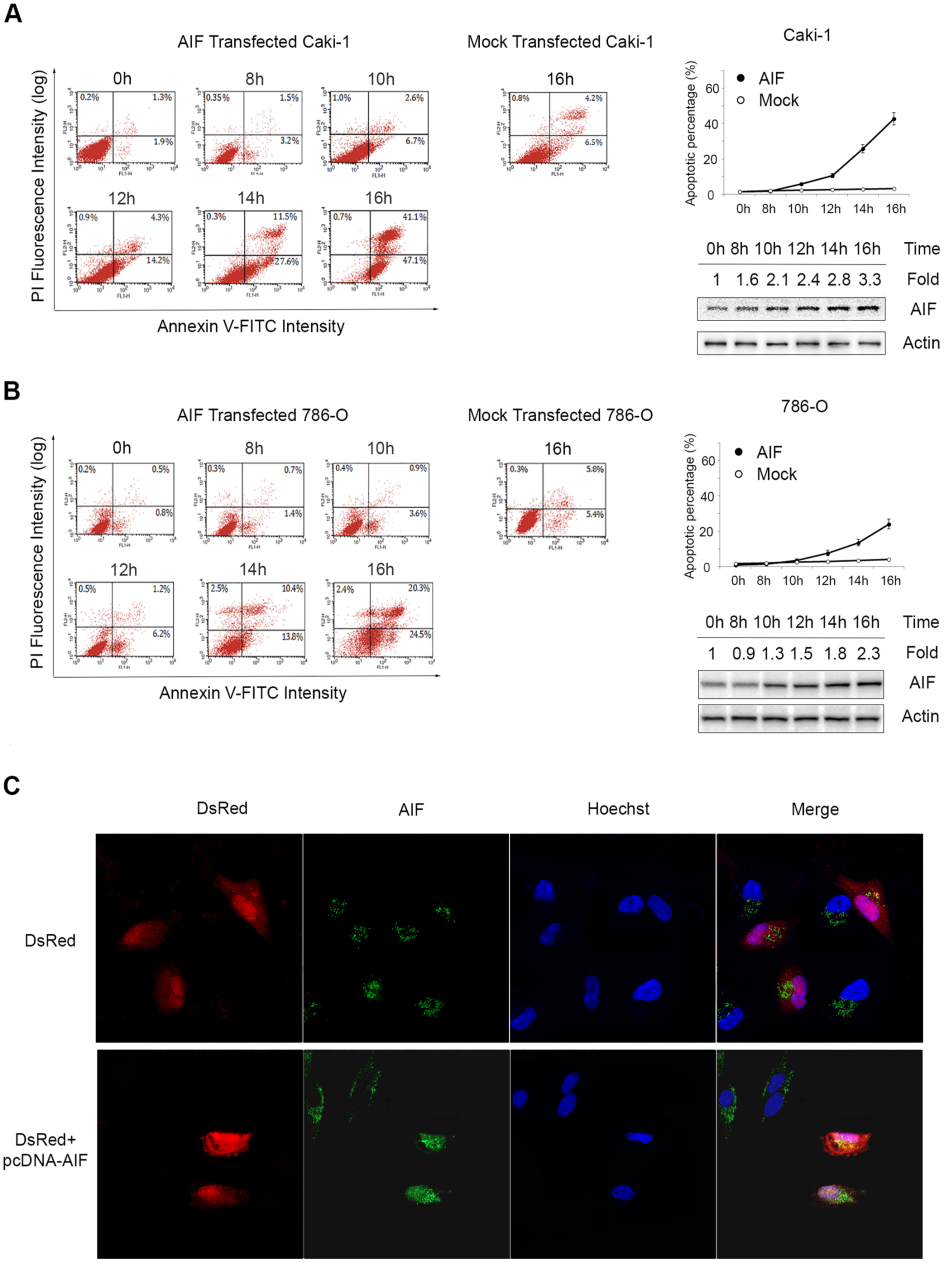
Forced AIF expression induced apoptosis in RCC cells. (A) Caki-1 or (B) 786-O cells were transfected with *pcDNA3.1* ***-*** *AIF* or vector only. Cells were harvested at various time points and apoptosis was evaluated by FCM with Annexin V-FITC/PI double stain kit. Apoptotic cell percentage is shown in the line chart. AIF expression was analyzed by Western blot. Relative folds of AIF expression were noted on the upper part of the panel. (C) Caki-1 cells were transfected with *pDsRed* (control) or *pDsRed*+*pcDNA3.1-AIF* (1∶10). AIF was detected by immunofluorescence stain. Cells with AIF overexpression underwent apoptosis (nuclear break down and nuclear localization of AIF).

To study whether forced expression of AIF affects tumor cell growth, *pcDNA3.1-AIF* was transiently transfected into 2 RCC cell lines, Caki-1 and 786-O. As shown in Figure 3A and 3B, AIF overexpression induced massive apoptosis in both Caki-1 and 786-O cells. Indeed, most transfected cells were dead and detached from culture flasks within 24 h after transfection. As determined by immunofluorescence stain, Caki-1 cells overexpressing *pcDNA3.1-AIF* had AIF signals in nuclei, consistent with its role in apoptosis (Fig. 3C).

### AIF interacts with STK3 and induces STK3 phosphorylation in RCC cell line

**Figure 4:**
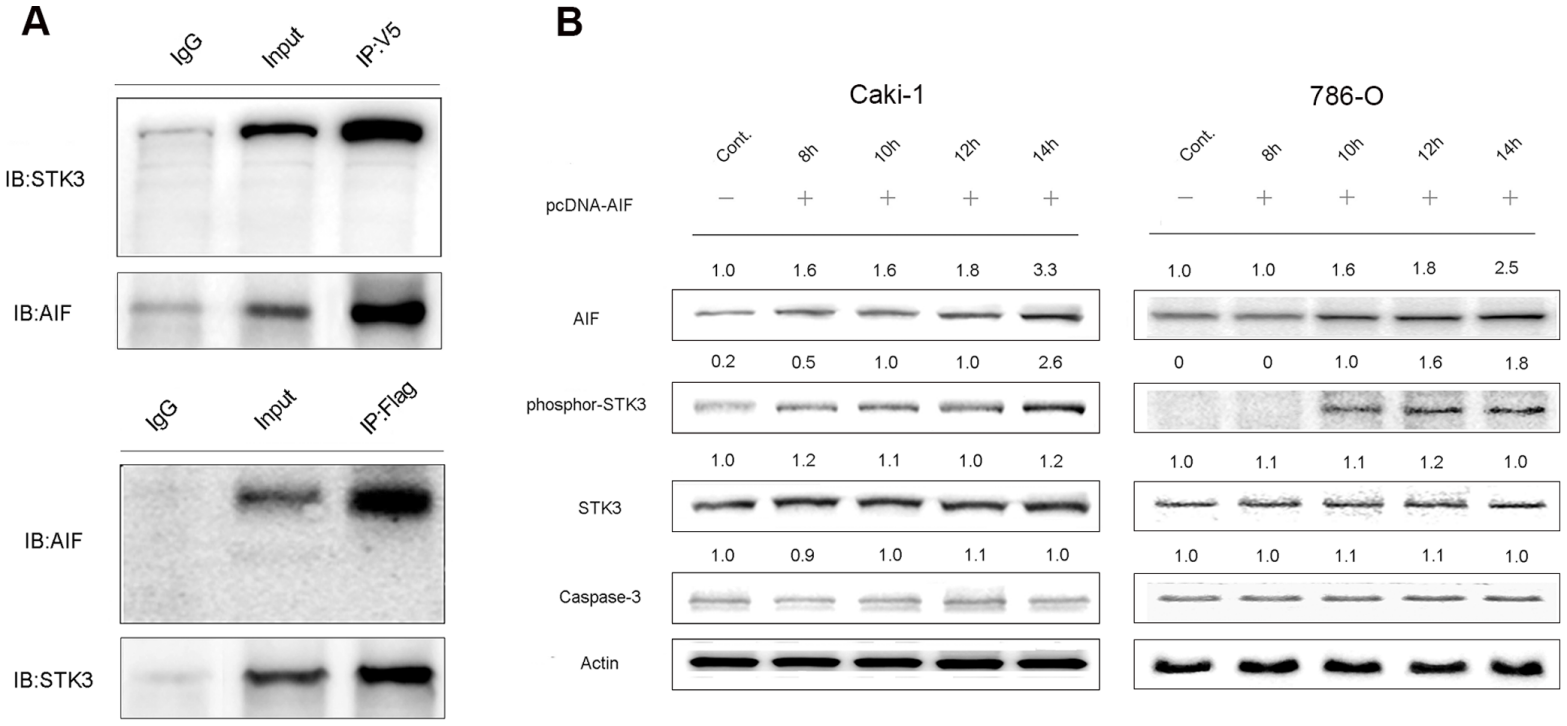
AIF interacted with STK3. (A) Immunorecipitation was performed in Caki-1 cells expressing *pcDNA3.1-AIF-V5* or *pCMV-flag-STK3*; Endogenous STK3 co-precipitated with AIF-V5, FLAG-STK3 was pulled down with endogenous AIF. (B) Caki-1 or 786-O cells transfected with *pcDNA3.1-AIF* were harvested at various time points and western blots were performed with antibodies against AIF, phosphor-STK3, STK3, caspse-3 and actin. Relative folds are noted on each panel. AIF overexpression induced significant increase of STK3 phosphorylation at Thr180.

To study the mechanisms of AIF-induced apoptosis in RCC, we performed a GST-AIF pull down assay using Caki-1 cell lysates. Pull down products were separated in a SDS-PAGE gel and proteins were identified by mass spectrometry. One of the AIF-interacting proteins was STK3 (Fig. S3). To confirm this result, *pcDNA3.1-AIF-V5* was expressed in Caki-1 cells, and immunoprecipitation was performed with anti-V5 antibody. Endogenous STK3 was detected by western blot in the co-precipitation of AIF-V5. A similar study of Caki-1 cells overexpressing FLAG-tagged STK3 showed co-precipitation of STK3-FLAG with endogenous AIF (Fig. 4A).

To study whether AIF expression affects STK3 activation, Caki-1 and 786-O cells were transfected with *pcDNA3.1-AIF* construct. AIF expression increased from 8 h to 14 h after transfection. As AIF increased, a significant increment of STK3 phosphorylation at Thr180 was detected, while the total STK3 did not change. No significant caspase-3 activation was observed during these time points (Fig. 4B).

## Discussion

AIF was initially identified as a candidate tumor suppressor because of its role in apoptosis. Upon an apoptotic signal, AIF translocates from mitochondria to nucleus where it triggers caspase-independent programmed cell death. Paradoxically, AIF also plays a role in cell survival. It has been reported that AIF is required to maintain the mitochondrial respiratory complex I. Moreover, AIF is a free radical scavenger that prevents apoptosis induced by reactive oxygen species [15], [34]. In fact, the role of AIF on cell death may be cell type-specific. For example, AIF gene silencing protects renal tubular epithelial cells against cisplatin-induced cell death [35]. However, AIF deficient human colon carcinoma cells are more sensitive to peroxide- and drug-induced apoptosis than the wild type counterparts [16]. AIF is predominantly overexpressed in several tumors including colon and squamous cell carcinomas [36], [37]. In this study, we reported that AIF expression was considerably higher in normal kidney as compared to the remaining 18 tissue types studied. Furthermore, significant AIF downregulation was detected in most kidney tumors. These studies suggest a tissue-specific role of AIF in tumorigenesis. AIF downregulation was also detected in a case of adenoma, which indicates that AIF downregulation is an early event in kidney tumor development. Our studies show that both genomic deletion and promoter methylation cause AIF downregulation in RCC, although intragenic mutations were not identified.

The profiling of DNA methylation in AIF promoter region using bisulfite sequencing showed a complex pattern including frequent methylcytosines in non-CpG sequences in RCC. Furthermore, Caki-1 cells had a CpT methylation, and demethylation by 5-aza-dC enhanced AIF expression significantly. Although the functional significance of non-CpG methylation is not completely understood, increasing data have implied the correlations between non-CpG methylation and tissue-specific gene expression. For example, non-CpG methylation is reported to be more prevalent in wild-type murine ES Cells than in tissues [38], and studies of murine NF1 gene have revealed non-CpG methylation in the oocyte and the maternally derived allele of the 2-cell embryo [39]. Recently, cytosine hypermethylation was detected in peroxisome proliferator-activated receptor γ coactivator-1 α (*PGC-1α*) promoter region in diabetic specimens, and the majority of the methylated cytosines were non-CpG methylation. It has also been reported that human myotubes treated with tumor necrosis factor-α (TNF-α) have acute increase of non-CpG methylation level in PGC-1α promoter [40]. These findings suggest non-CpG methylation may carry out complex biological function in cells.

AIF induces DNA fragmentation and chromatin condensation when entering the nucleus. However, the exact mechanisms are not completely understood. It is well accepted that AIF does not possess nuclease activity, and the DNA fragmentation induced by AIF depends on its DNA binding capability. In an *in vitro* cell-free system, AIF alone is capable of inducing chromatin condensation and DNA loss in the isolated nuclei [41], [42]. However, AIF has no effect on preheated nuclei. This evidence suggests that other nuclear factors are involved in AIF-induced chromatin condensation [43]. Three nuclear proteins have been reported to be associated with AIF: Endonuclease G, Cyclophilin A, and H2AX. H2AX is a major component of chromatin and plays a role in assembling DNA repair complex when DNA damage occurs. The interaction between AIF and H2AX is believed to play a role in caspase-independent DNA fragmentation [13]. AIF binds to H2AX only when its ser139 is phosphorylated. Interestingly, STK4, a STK3 family member, is capable of phosphorylating ser139 of H2AX [44]. Our study showed that AIF binds to STK3, leading to increasing STK3 activity. We are currently studying the possibility that AIF-induced STK3 activation phosphorylates H2AX in kidney cells that, in return, binds to AIF to induce apoptosis.

Kidney cancer is one of the most common cancers, and several types of kidney tumors are more common in male. For example, male-to-female ratios for CCRCC, Bellini duct carcinoma and medullary carcinoma are approximately 2∶1. Male patients also have higher possibility of metastatic spread and worse prognosis in CCRCC [26]. Interestingly, AIF is located on chromosome X. Whether AIF is capable of escaping chromosome X inaction and whether leaking AIF expression contributes to protection from malignant transformation in female patients are probably worth further evaluation.

In summary, our finding that AIF is frequently downregulated in RCC suggests that loss of AIF may be involed in RCC development. The interaction between AIF and STK3 also reveals a novel role of AIF in regulating programmed cell death. Targeting the AIF signaling pathway therefore may be a viable option for the treatment of RCC patients.

## Supporting Information

Figure S1 Immunohistochemical analysis of paraffin-embedded human kidney, using AIF (AB1998) antibody (A) and negative control (B).
(TIF)

Figure S2 Sequencing results of AIF promoter methylation from RCC tumor specimens. Each small figure shows relative position of methylated cytosines and the specimens it belongs to.
(TIF)

Figure S3 (A) GST pull-down assay using GST-AIF fusion protein and Caki-1 cell lysate. Arrow showed the band corresponding to STK3. (B) Massspectrometry results of the STK3 band.
(TIF)

Table S1 Characteristics of renal cell carcinoma tissue.
(DOC)

Table S2 BSP primers for AIF promoter.
(DOC)

Table S3 Clinicopathologic findings in RCC specimens (n = 51).
(DOC)

Table S4 Summary of AIF expression and clinical pathologic features in CCRCC.
(DOC)

Table S5 Primers used in amplification of AIF exons.
(DOC)
