# Supplementary figures and images for: AIF Downregulation and Its Interaction with STK3 in Renal Cell Carcinoma
Source: PLoS One. 2014 Jul 3;9(7):e100824. doi: 10.1371/journal.pone.0100824 (PMC4081115; doi:10.1371/journal.pone.0100824)

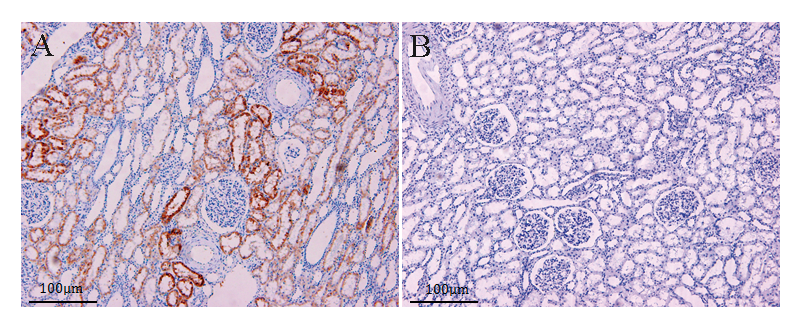

Supplement: Figure S1 — Immunohistochemical analysis of paraffin-embedded human kidney, using AIF (AB1998) antibody (A) and negative control (B). (TIF) [file pone.0100824.s001.tif]

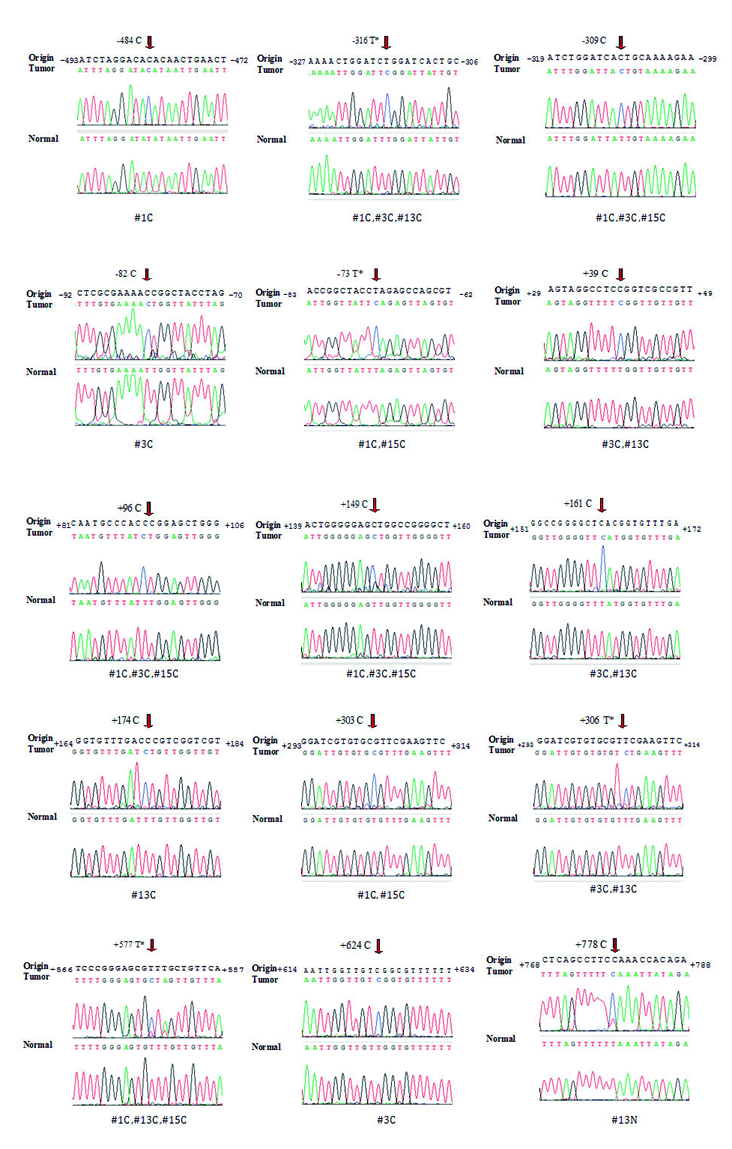

Supplement: Figure S2 — Sequencing results of AIF promoter methylation from RCC tumor specimens. Each small figure shows relative position of methylated cytosines and the specimens it belongs to. (TIF) [file pone.0100824.s002.tif]

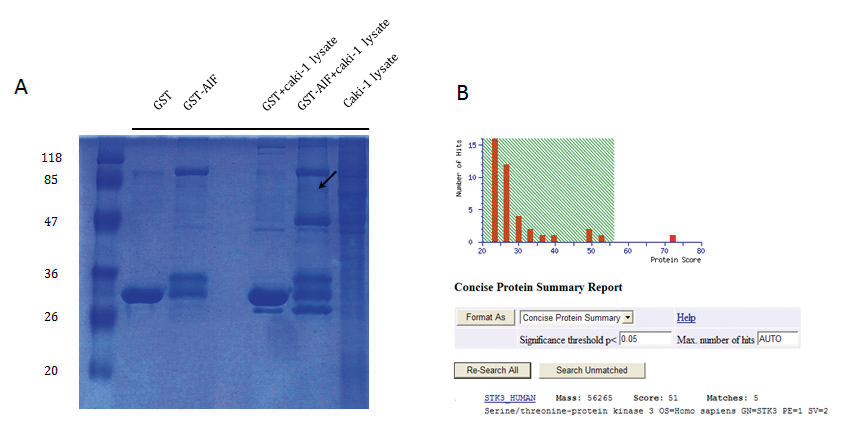

Supplement: Figure S3 — (A) GST pull-down assay using GST-AIF fusion protein and Caki-1 cell lysate. Arrow showed the band corresponding to STK3. (B) Massspectrometry results of the STK3 band. (TIF) [file pone.0100824.s003.tif]
