# Supplementary material for: AIF Downregulation and Its Interaction with STK3 in Renal Cell Carcinoma
Source: PLoS One. 2014 Jul 3;9(7):e100824. doi: 10.1371/journal.pone.0100824 (PMC4081115; doi:10.1371/journal.pone.0100824)
Supplement: Table S1 — Characteristics of renal cell carcinoma tissue. (DOC) [file pone.0100824.s004.doc]

Table S1. Characteristics of renal cell carcinoma tissue

| Variable | Categorization | n analyzable | % |
| --- | --- | --- | --- |
| Clinicopathologic data |  |  |  |
| Tumor stage |  |  |  |
|  | T1 | 42 | 82.4 |
|  | T2 | 2 | 3.9 |
|  | T3 | 7 | 13.7 |
| Lymph node status |  |  |  |
|  | N0 | 49 | 96.1 |
|  | N2 | 2 | 3.9 |
| Metastases status |  |  |  |
|  | M0 | 44 | 92.2 |
|  | M1 | 7 | 7.8 |
| Classification |  |  |  |
|  | CCRCC* | 45 | 88.2 |
|  | PRCC** | 4 | 7.8 |
|  | ChRCC***  renal oncocytoma | 1  1 | 2.0  2.0 |
| Gender |  |  |  |
|  | male | 29 | 56.9 |
|  | female | 22 | 43.1 |

* CCRCC=Clear Cell Renal Cell Carcinoma, ** PRCC=Papillary Renal Cell Carcinoma; *** ChRCC= Chromophobe Renal Cell Carcinoma
