# Supplementary material for: AIF Downregulation and Its Interaction with STK3 in Renal Cell Carcinoma
Source: PLoS One. 2014 Jul 3;9(7):e100824. doi: 10.1371/journal.pone.0100824 (PMC4081115; doi:10.1371/journal.pone.0100824)
Supplement: Table S2 — BSP primers for AIF promoter. (DOC) [file pone.0100824.s005.doc]

Table S2. BSP primers for AIF promoter

| **primer** | **primer sequence** | **product (bp)** |
| --- | --- | --- |
| **Part 1** |  |  |
| P1/F1 | 5′-TGAAAGTTTTGTTTAGTAGATTTTTTTTTTTAGAAAG-3′ | 355 |
| P1/F2 | 5′-AAGTATAGAAATTATAGTAGGAGATTGTGTGTATTTG-3′ |
| P1/R | 5′-TAATAACRAAAACTAATAACAACATCTCACAACAAC-3′ |
| **Part 2** |  |  |
| P2/F1 | 5′-TTGAATAGGTTTTTTAATTYGTTT-3′ | 504 |
| P2/F2 | 5′-TTAATTCGTTTAGTTTAATATTGTG-3′ |
| P2/R | 5′-CCAAACCCTCGAACTTAAAAATTACCTAAAATAAATC-3′ |
| **Part 3** |  |  |
| P3/F | 5′-TGTTTTAGGTTTTTTGTGTTTTGTAG-3′ | 317 |
| P3/R1 | 5′-TAAACCAATTCTCTAAATATCTAATACACAACC-3′ |
| P3/R2 | 5′-AAAATCTCAATCAATAAATAACAAAATCTACTTAACC-3′ |
