# Supplementary material for: AIF Downregulation and Its Interaction with STK3 in Renal Cell Carcinoma
Source: PLoS One. 2014 Jul 3;9(7):e100824. doi: 10.1371/journal.pone.0100824 (PMC4081115; doi:10.1371/journal.pone.0100824)
Supplement: Table S3 — Clinicopathologic findings in RCC specimens (n = 51). (DOC) [file pone.0100824.s006.doc]

Table S3. Clinicopathologic findings in RCC specimens (n=51)

| Case  # | Age | Sex | Size  (cm) | Local  invasion | Perineural  invasion | Lymphnode  metastasis | Distant  metastasis | WHO  classification | Pathology  classification | AIF expression | |
| --- | --- | --- | --- | --- | --- | --- | --- | --- | --- | --- | --- |
|  |  |  |  |  |  |  |  |  |  | NA tissue | Tumor tissue |
| 1 | 38 | M | 3 | NO | NO | NO | NO | T1N0M0 | CCRCC | ++ | − |
| 2 | 41 | F | 6 | NO | NO | NO | NO | T1N0M0 | CCRCC | ++ | − |
| 3 | 65 | M | 4.5 | NO | NO | NO | NO | T1N0M0 | CCRCC | ++ | − |
| 4 | 59 | F | 3.5 | NO | NO | NO | NO | T1N0M0 | CCRCC | ++ | − |
| 5 | 52 | F | 1.5 | NO | NO | NO | NO | T1N0M0 | CCRCC | +++ | − |
| 6 | 68 | F | 5 | NO | NO | NO | NO | T1N0M0 | CCRCC | ++ | − |
| 7 | 43 | M | 3 | NO | NO | NO | NO | T1N0M0 | CCRCC | +++ | − |
| 8 | 53 | M | 4 | NO | NO | NO | NO | T1N0M0 | PRCC | ++ | − |
| 9 | 33 | M | 1.5 | NO | NO | NO | NO | T1N0M0 | CCRCC | +++ | − |
| 10 | 41 | F | 10 | NO | NO | NO | NO | T2N0M0 | CCRCC | ++ | − |
| 11 | 56 | F | 3 | NO | NO | NO | NO | T1N0M0 | CCRCC | ++ | − |
| 12 | 57 | F | 2 | NO | NO | NO | NO | T1N0M0 | PRCC | +++ | − |
| 13 | 37 | M | 6 | NO | NO | NO | NO | T1N0M0 | CCRCC | ++ | − |
| 14 | 53 | M | 5 | NO | NO | NO | NO | T1N0M0 | CCRCC | +++ | − |
| 15 | 67 | M | 4 | NO | NO | NO | NO | T1N0M0 | CCRCC | +++ | − |
| 16 | 55 | F | 2 | NO | NO | NO | NO | T1N0M0 | CCRCC | ++ | − |
| 17 | 62 | M | 2.5 | YES | YES | NO | NO | T4N0M1 | CCRCC | ++ | − |
| 18 | 43 | M | 5 | NO | NO | NO | NO | T1N0M0 | CCRCC | ++ | − |
| 19 | 49 | M | 5 | NO | NO | NO | NO | T1N0M0 | CCRCC | +++ | − |
| 20 | 40 | M | 4 | YES | YES | NO | NO | T4N0M1 | CCRCC | +++ | − |
| 21 | 46 | M | 2.5 | NO | NO | NO | NO | T1N0M0 | CCRCC | ++ | − |
| 22 | 57 | F | 4.5 | NO | NO | NO | NO | T1N0M0 | oncocytoma | +++ | − |
| 23 | 46 | M | 6 | NO | NO | NO | NO | T1N0M0 | CCRCC | ++ | − |
| 24 | 53 | F | 3 | NO | NO | NO | NO | T1N0M0 | CCRCC | ++ | − |
| 25 | 68 | F | 3 | NO | NO | NO | NO | T1N0M0 | CCRCC | ++ | − |
| 26 | 53 | M | 3 | NO | NO | NO | NO | T1N0M0 | CCRCC | + | + |
| 27 | 49 | M | 6 | NO | NO | NO | NO | T1N0M0 | CCRCC | +++ | − |
| 28 | 53 | M | 5 | NO | NO | NO | NO | T1N0M0 | CCRCC | +++ | − |
| 29 | 38 | M | 3 | NO | NO | NO | NO | T1N0M0 | PRCC | +++ | ++ |
| 30 | 76 | F | 3.5 | NO | NO | NO | NO | T1N0M0 | CCRCC | ++ | − |
| 31 | 54 | M | 4 | NO | NO | NO | NO | T1N0M0 | CCRCC | ++ | + |
| 32 | 82 | F | 5 | NO | NO | NO | NO | T1N0M0 | CCRCC | ++ | + |
| 33 | 66 | F | 5 | NO | NO | NO | NO | T1N0M0 | CCRCC | ++ | − |
| 34 | 39 | M | 5 | NO | NO | NO | NO | T1N0M0 | CCRCC | +++ | + |
| 35 | 49 | M | 1.5 | NO | NO | NO | NO | T1N0M0 | CCRCC | +++ | − |
| 36 | 44 | F | 7 | YES | YES | NO | NO | T4N0M1 | ChRCC | +++ | ++ |
| 37 | 62 | M | 12.5 | YES | YES | YES | YES | T4N1M1 | CCRCC | ++++ | ++ |
| 38 | 54 | M | 5 | NO | NO | NO | NO | T1N0M0 | CCRCC | NA | − |
| 39 | 53 | F | 5 | NO | NO | NO | NO | T1N0M0 | CCRCC | NA | − |
| 40 | 57 | M | 2.5 | NO | NO | NO | NO | T1N0M0 | CCRCC | NA | − |
| 41 | 28 | M | 4 | NO | NO | NO | NO | T1N0M0 | CCRCC | NA | − |
| 42 | 57 | F | 3 | NO | NO | NO | NO | T1N0M0 | CCRCC | NA | − |
| 43 | 68 | F | 2 | YES | YES | NO | NO | T4N0M1 | CCRCC | NA | − |
| 44 | 69 | M | 3 | NO | NO | NO | NO | T1N0M0 | PRCC | NA | − |
| 45 | 78 | F | 5 | YES | YES | NO | NO | T4N0M1 | CCRCC | NA | − |
| 46 | 66 | F | 4 | NO | NO | NO | NO | T1N0M0 | CCRCC | NA | − |
| 47 | 59 | M | 8 | NO | NO | NO | NO | T2N0M0 | CCRCC | NA | − |
| 48 | 62 | M | 4 | NO | NO | NO | NO | T1N0M0 | CCRCC | NA | − |
| 49 | 57 | F | 5 | YES | YES | NO | NO | T4N0M1 | CCRCC | NA | − |
| 50 | 58 | M | 4 | NO | NO | NO | NO | T1N0M0 | CCRCC | NA | + |
| 51 | 70 | M | 3.5 | NO | NO | NO | NO | T1N0M0 | CCRCC | NA | − |
