# Supplementary material for: AIF Downregulation and Its Interaction with STK3 in Renal Cell Carcinoma
Source: PLoS One. 2014 Jul 3;9(7):e100824. doi: 10.1371/journal.pone.0100824 (PMC4081115; doi:10.1371/journal.pone.0100824)
Supplement: Table S4 — Summary of AIF expression and clinical pathologic features in CCRCC. (DOC) [file pone.0100824.s007.doc]

Table S4. Summary of AIF expression and clinical pathologic features in CCRCC

| Pathological  parameters | | total | | AIF expression in RCC (n) | | | *P* value |
| --- | --- | --- | --- | --- | --- | --- | --- |
|  |  |  |  | −,+/− | | + ~ +++ |  |
| Age | | | | | | | |
| < 60 | 31 | | 27 | | 4 | | > 0.05 |
| ≥60 | 14 | | 12 | | 2 | |  |
| Gender | | | | | | | |
| male | 37 | | 32 | | 5 | | > 0.05 |
| female | 18 | | 17 | | 1 | |  |
| Size of tumor | | | | | | | |
| <5cm | 27 | | 24 | | 3 | | > 0.05 |
| ≥5cm | 18 | | 15 | | 3 | |  |
| Metastasis | | | | | | | |
| No | 39 | | 37 | | 5 | | > 0.05 |
| Yes | 6 | | 5 | | 1 | |  |
