# Supplementary material for: AIF Downregulation and Its Interaction with STK3 in Renal Cell Carcinoma
Source: PLoS One. 2014 Jul 3;9(7):e100824. doi: 10.1371/journal.pone.0100824 (PMC4081115; doi:10.1371/journal.pone.0100824)
Supplement: Table S5 — Primers used in amplification of AIF exons. (DOC) [file pone.0100824.s008.doc]

Table S5. Primers used in amplification of AIF exons

| **exon** | **primer sequence** | **product (bp)** |
| --- | --- | --- |
| 1 | F/AIF E1: 5′-GGGCTCACGGTGTTTGAC-3′ | 259 |
|  | R/AIF E1: 5′-GTAGAGGGCTGCAAGGCACAG-3′ |  |
| 2 | F/AIF E2: 5′-GGCAATAAGTCTTGTACAGTG-3′ | 315 |
|  | R/AIF E2: 5′-AAAGCAGAATACTATCGAGTC-3′ |  |
| 3 | F/AIF E3: 5′-GTATGAGCTAGCTGTATGGTG-3′ | 252 |
|  | R/AIF E3: 5′-CATTGCTTCTACAAGACATCC-3′ |  |
| 4 | F/AIF E4: 5′-TATGTCTGAAGGTACTGATTG-3′ | 198 |
|  | R/AIF E4: 5′-CCTATTCTCTGTGCTTAGCTG-3′ |  |
| 5 | F/AIF E5: 5′-CAGTGGCAAAGAATCATCTGAG-3′ | 202 |
|  | R/AIF E5: 5′-AAACATGCACCTTACCCTGGC-3′ |  |
| 6 | F/AIF E6: 5′-GGAAGCTGATAACAGTTCCAC-3′ | 247 |
|  | R/AIF E6: 5′-ATGGCAGGACAGACATAAATG-3′ |  |
| 7 | F/AIF E7: 5′-GAATTTGATGTGAATTATATCTC-3′ | 244 |
|  | R/AIF E7: 5′-CCATAGAGAAGGCTGGACTC-3′ |  |
| 8 | F/AIF E8: 5′-GCTAAGTGAAGAGGGCTCC-3′ | 250 |
|  | R/AIF E8: 5′-GGGCACTTGGGGACTGCAAG-3′ |  |
| 9 | F/AIF E9: 5′-GGACTAAACTGATGTGATCC-3′ | 230 |
|  | R/AIF E9: 5′-CTCTTCCTACTGATCCTGCC-3′ |  |
| 10 | F/AIF E10: 5′-CTGCTGCTCCTTTACTTCTCTTC-3′ | 256 |
|  | R/AIF E10: 5′-GCGAAGTTTGCCTTAGGTCAC-3′ |  |
| 11 | F/AIF E11: 5′-GTGATAGCCTTGTAGGCTCAC-3′ | 192 |
|  | R/AIF E11: 5′-GGCAAGGGGAGTGGAGAACTG-3′ |  |
| 12 | F/AIF E12: 5′-CCTGTAGGCATAAATGGAAAC-3′ | 259 |
|  | R/AIF E12: 5′-CAGACTTGTTCACAGGCTAG-3′ |  |
| 13 | F/AIF E13: 5′-ATCATGTGTTCATGTTATGTA-3′ | 240 |
|  | R/AIF E13: 5′-GTGTTATGGTCCTAGAGATACTG-3′ |  |
| 14 | F/AIF E14: 5′-ATGTGCTACCGTGTCATTCC-3′ | 280 |
|  | R/AIF E14: 5′-CTTGTTCAGGAGAATCTGGAC-3′ |  |
| 15 | F/AIF E15: 5′-CCAAGCTCATAATAACTGAAG-3′ | 365 |
|  | R/AIF E15: 5′-CGATGAAGTTACAGGAATGTTTC-3′ |  |
| 16 | F/AIF E16: 5′-CCTGAACGCAATGGAGTAAG-3′ | 410 |
|  | R/AIF E16: 5′-CAATTTGCTTGCTTTCTAATATGC-3′ |  |
